# Development of a novel approach for restoration of the meniscus using silk-elastin in a rabbit meniscus injury model

**DOI:** 10.1186/s12891-024-07675-9

**Published:** 2024-07-15

**Authors:** Tadashi Inoue, Toshiya Kano, Tomoyuki Nakasa, Masakazu Ishikawa, Keiichiro Inoue, Shingo Kawabata, Shigeru Miyaki, Naosuke Kamei, Nobuo Adachi

**Affiliations:** 1 Department of Orthopaedic Surgery, Graduate School of Biomedical and Health Sciences, Hiroshima University, 1-2-3 Kasumi, Minami-Ku, Hiroshima, 734-8553 Japan; 2 Department of Artificial Joints and Biomaterials, Graduate School of Biomedical and Health Sciences, Hiroshima University, Hiroshima, Japan; 3 Medical Center for Translational and Clinical Research, Hiroshima University Hospital, Hiroshima, Japan; 4 Sanyo Chemical Industries Ltd, Kyoto, Japan

**Keywords:** Meniscal injury, Silk-elastin, Meniscal repair, Rabbit model

## Abstract

**Background:**

Limited healing potential of the meniscus remains a burden for the successful repair of meniscus injuries in the orthopaedic fields. Silk-elastin (SE) is a novel recombinant protein with favorable properties for wound healing. This proof-of-concept study aimed to investigate the therapeutic effect of silk-elastin in a rabbit meniscal defect model.

**Methods:**

A migration assay using rabbit meniscus and synovial cells with various concentrations of SE in a culture medium was conducted to investigate the mechanism of meniscal healing by SE. Additionally, cylindrical defects with a 1.5 mm diameter were created at the anterior horn of the medial meniscus of rabbits. The animals were divided into three groups: 1) the Blank group; defect only, 2) the Col I group; implantation of type I atelocollagen sponge, and 3) the SE group; implantation of SE (150 mg/ml) sponge. Whole medial menisci were harvested at 4, 8, 12, and 24 weeks after surgery. Histological analyses including immunohistochemical staining were performed to assess meniscal healing.

**Results:**

In vitro study, Migration assay demonstrated a significantly higher number of migrated cells only in synovial cells. Especially, the SE concentration of 10 µg/mL demonstrated the highest number of migrated cells compared with other concentrations. In vivo study, the SE group exhibited significantly higher Ishida scores than other groups at all time points. Furthermore, the SE group showed higher synovial coverage scores than the Col I group at 4 and 8 weeks. Immunohistochemical staining demonstrated higher type II collagen staining in the SE group compared to other groups at 12 weeks. Implanted SE was efficiently replaced by safranin-O staining positive tissue within 8 weeks.

**Conclusions:**

SE could effectively repair a meniscal defect by inducing coverage of synovial cells. SE has the potential to be a useful material for meniscal repair.

## Introduction

Meniscal injuries, most frequently tears, are a common and important source of knee dysfunction and disability, with an annual incidence of 66 per 100,000 people [1]. Partial meniscectomy is recognized as a well-tolerated treatment option [2]. However, meniscectomy has been reported to accelerate the degeneration of articular cartilage due to alteration of stress distribution, which leads to osteoarthritis of the knee joint [3]. Despite these clinical concerns, meniscectomy is indicated as the first-line treatment for many meniscal tears and is performed at a rate 5 to 24 times higher than meniscal repair [4–6]. The poor reparative capacity of the meniscus, especially in the avascular inner zone of the meniscus, and the difficulty of meniscus suture techniques are among the reasons for this situation. Therefore, various attempts have been made to preserve the meniscus.

Like any other tissue regeneration strategy, meniscus tissue engineering also has three major essentials such as cell source, biomolecules, and biomaterials [7]. Since recent developments in science and technology have led to significant advances in materials, we focused on the application of biomaterials for meniscus regeneration. Scaffolds for meniscus tissue engineering are made up of natural, synthetic, or composite polymers [8]. The physicochemical requirements of ideal scaffolds are easy processability to desired shapes, adequate mechanical strength, porous (large surface-to-volume ratio), biodegradable, and biocompatible so that it can provide an adequate environment for cellular proliferation and extracellular matrix synthesis [9].

Out of the variety of scaffolds for meniscal repair used in vivo, only two of these, CMI® and Actifit® were introduced into clinical practice. The first one is a collagen-based implant released in the late 90ies, whereas the latter one is a polyurethane-based scaffold which reached the market a few years later. Both are cell-free scaffolds which aim at promoting regeneration of meniscal fibrocartilage by stimulating resident stromal cells from adjacent tissues, especially synovium. Safety and efficacy data of these scaffolds for meniscal healing and inherent cartilage protection were reported [10–21]. However, it takes time for the tissue to be replaced and may fail due to mechanical stress before it is replaced, a complete meniscal healing has been seen only occasionally and we are still far from reaching it with the current technologies. Accordingly, new scaffolds should be manufactured with appropriate biomechanical properties that match those required for normal joint function while new tissue is being formed. Furthermore, they should be easily replaced by regenerated tissue [22].

Silk-elastin (SE) is a protein generated by recombinant DNA technology with relevant genes of silkworm fibroin and human elastin to generate peptide repeats of the silk-fibroin derived sequence (GAGAGS) and elastin-derived sequence (GVGVP) [23]. Previously, it was reported that SE hydrogels have the potential to accelerate wound healing in decubitus ulcers in diabetic mice and SE activates the cell migration ability of mouse fibroblasts and macrophages [24, 25]. SE has not only a high cell affinity and rich elasticity but also another unique feature of its variety of form, from gel to sponge. With modification of manipulation, the sponge form with various stiffness to tolerate mechanical force could be obtained. Previously, it was reported that SE sponges can manage and promote regeneration of bronchial epithelium in Beagle dogs and is safe for chronic skin ulcers in human [26, 27]. These biological and mechanical properties of SE will have favorable effects and could be a novel solution for meniscus repair. In this study, SE was focused as a scaffold on acceleration meniscal repair using its sponge form. The purpose of this study is to investigate the therapeutic effect of SE in rabbit meniscal defect models. We hypothesized that 1) SE would induce migration of cells (meniscal cells and synovial cells), and 2) SE would induce good tissue remodeling when implanted into the injured meniscus.

## Materials and methods

### Study design

The study was carried out strictly in accordance with the ARRIVE guidelines and the Guidelines for Animal Experimentation at our institution. The experimental protocol was approved by the Ethics Review Committee for Animal Experimentation of the Graduate School of Biomedical Sciences, Hiroshima University (approval number: A17-103). To investigate mechanism of action of SE, transwell migration assay was carried out. The meniscal and synovial cells from Japanese white rabbit (2.5 to 3.0 kg, Kitayama Labes Co., Ltd., Nagano, Japan) were seeded onto transwell inserts in 12-well plates with culture medium containing various concentration of SE. After 12 h, migrated cells were stained with 4′,6-diamidino-2-phenylindole and counted under fluoromicroscope. In vivo study consisted of 2 treatment groups and a control group. A cylindrical punch defect was created in the avascular zone at the anterior horn of medial meniscus in Japanese white rabbit (2.5–3.0 kg). To eliminate the influence of gender differences, the rabbits were gendered as male. Two treatment approaches comprised two different materials, *i.e.* type I collagen or SE sponges (*n* = 6 in each group). In a control group (*n* = 6), only cylindrical defects in medial meniscus were created. Rabbits were sacrificed 4, 8, 12, and 24 weeks after surgeries.

### Transwell migration assay

The migration ability of rabbit meniscal cell and rabbit synovial cells in SE was investigated using a migration assay. Synovial and meniscus tissues were collected from the rabbit’s bilateral knee joints (*n* = 2), and rinsed 3 times in Phosphate buffered saline to remove blood and fat, cut into pieces not exceeding 1 mm^3^. Synovial tissues were placed dots in the bottom of the culture dishes and were cultured in Dulbecco’s Modified Eagle’s Medium (FUJIFILM Wako Pure Chemical Co., Osaka, Japan) supplemented with 10% fetal bovine serum (Thermo Fisher Scientific Co., Ltd., Tokyo, Japan). When the outgrowth cells reached 80–90% confluence, synovial tissues were removed and 0.25% trypsin was used to passage cells, and 3–5 generations were used for in vitro study. Meniscus cells were isolated using 1% collagenase I (Worthington Biochemical Co., Lakewood, NJ) for 4 h at 37 °C in an incubator with 5% CO^2^. After centrifugation at 1,500 rpm for 5 min, cell pellets were suspended in a medium and plated in the culture dish. Mediums in which different amounts of SE were dissolved (SEM) were prepared for the purpose of investigating whether the migration ability changed depending on the concentration. The cultivation prepared with serum-free medium and 10% fetal bovine serum supplemented medium were used for positive control. The manufactured kit (CytoSelect Boyden Chamber Cell Migration/Invasion Assay, Funakoshi Co., Ltd., Tokyo, Japan) was used in the experiment. The cell suspension is added to the upper chamber and incubated for 12 h. Through the polycarbonate membrane, migrating cells adhere to the bottom surface of the membrane and non-migrating cells remain in the upper chamber. After removing the non-migrating cells, the migrating cells were stained with 4′,6-diamidino-2-phenylindole. Migrated cells were imaged with a fluorescence microscope (BZ-9000, Keyence, Osaka, Japan) and quantified. Synovial and meniscus cells were collected from another two rabbit’s bilateral knee joints (*n* = 4) and migration assay were carried out using for each cell in duplicate. The average value for a total of four experiments was calculated.

### Surgical procedure

**Fig. 1:**
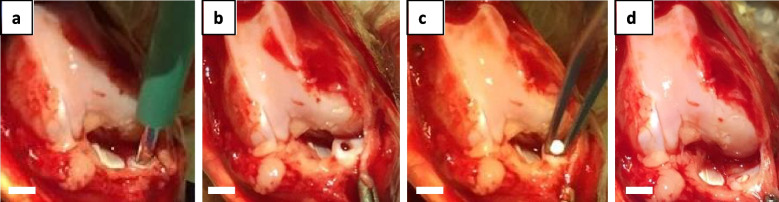
Transplantation of the silk elastin (SE) sponge into the meniscal defect. **A** Creating full thickness cylindrical defect with 1.5 mm diameter. **B** Meniscal defect at the anterior horn of the medial meniscus. **C** Transplantation of the SE sponge. **D** After transplantation. Bars; 5 mm

A total of 38 Japanese white rabbits weighing 2.5 to 3.0 kg were used in this study (Kitayama Labes Co., Ltd., Nagano, Japan). Surgical procedures were performed according to previously reports [28, 29]. Under general anesthesia using ketamine hydrochloride and xylazine, surgery was performed. Through a medial parapatellar approach, the patella was dislocated laterally and the medial meniscus was disclosed. Full thickness cylindrical defects with a 1.5 mm diameter were made in the avascular zone of the anterior horn of the medial meniscus by using a biopsy punch (Kai Medical, Gifu, Japan) (Fig. 1). The 72 defects from 36 rabbits were divided into 3 groups according to treatment as follows: 1) the Blank group (blank defect), 2) the Col I group (defect implantation with type I atelocollagen sponge (Mighty, Koken, Japan)), and 3) the SE group (defect implantation with SE sponge (150 mg/ml)). Since type II collagen is not commercially available in sponge or solid form, type I collagen, which is used clinically, was used as a control group. For SE implantation, SE was prepared as previously described [27, 30] and was provided by Sanyo Chemical, Kyoto, Japan. The concentration of silk-elastin in the sponges was 150 mg/mL considering hydrophilicity and cell migration ability. The device was 1.5 mm in diameter and 3 mm in thickness. The joint capsule and skin were sutured as separate layers in all groups. After surgery, all rabbits were returned to their cages and allowed to move freely without joint immobilization. Whole medial menisci were harvested at 4, 8, 12, and 24 weeks after surgery (*N* = 6 in each group). The two knees from a rabbit were created by the same surgical procedure as a Time 0 model of SE implantation, and the other two knees from another rabbit were used as a normal model.

### Histological analysis

After the whole medial meniscus was resected from the knee, radial sections were made at the anterior portion. The samples were embedded in paraffin and sectioned at a thickness of 4.5 µm, deparaffinized in xylene, dehydrated using an ethanol series, and stained with safranin-O / fast green and hematoxylin & eosin (HE). Histological sections stained with safranin-O / fast green were blinded and evaluated using Ishida scoring system which includes 3 assessment points: 1) reparative tissue with bonding, assessing whether the reparative tissues had bonded with the surrounded normal meniscus tissues, 2) the existence of fibrochondrocytes, and 3) stainability with safranin-O [4]. In order to enforce a more accurate assessment of the above three items, we modified the Ishida score so that 0% is 0, ~ 25% is 0.5, 25–50% is 1, 50–75% is 1.5, and 75–100% is 2. The total score was kept at 6, as a higher score means a better restoration was obtained. In addition, synovial cell migration toward the implanted sites in the Col I and the SE groups of HE-stained sections was evaluated using the synovial coverage scoring system based on that developed by Nakagawa et al. [31] (Table 1).

**Table 1:** Synovial coverage scoring for meniscus healing. The score is determined by the number of layers of synovial cells present in the lesion. The score is determined by the number of synovial cell layers on the surface of the implanted area

Definition	Score
The lesion is not covered by synovium	0
The lesion is covered by synovium with 1–2 synovial cell layers	1
The lesion is covered by synovium with 3–5 synovial cell layers	2
The lesion is covered by synovium with 6 or more synovial cell layers	3

### Immunohistochemical analysis

Immunohistochemical staining was carried out on a series of 5 um thick sections. The sections were pretreated with antigen retrieval reagent (Immunoactive; Matsunami Glass Ind) for 1 h followed by 0.3% H_2_O_2_ for 30 min, normal blocking serum for 30 min, and primary antibody against type I collagen (1:250 dilution; Novus Biologicals) and type II collagen (1:100 dilution; Daiichi Fine Chemical) overnight at 4 °C. On the next day, the sections were visualized using the avidin–biotin system (Vectastain Elite ABC Mouse IgG kit; Vector Laboratories Inc) and 3,3#-diaminobenzidine (Peroxidase Substrate Kit; Vector Laboratories Inc), according to the manufacturer’s instructions. The staining ratio of type I and II collagen was calculated as the collagen staining scores. Using Zellner's scoring system [32] as a reference, we assigned a staining rate of 0% to 0, ~ 25% to 1, 25–50% to 2, 50–75% to 3, and 75–100% to 4. For detection of SE localization, rabbit polyclonal antibody against SE (dilution, 1: 1000) was used and prepared sections were stained following the same protocol as described above.

### Statistical analysis

The results were expressed as means ± standard deviation. The Ishida scores and the collagen staining scores were analyzed with the Student–Newman–Keuls method. The number of migrated cells and the synovial coverage scores were analyzed with the Mann–Whitney test (Prism, GraphPad Software, MA, USA). *P* values < 0.05 were statistically significant.

## Results

### Cell migration to SE medium

**Fig. 2:**
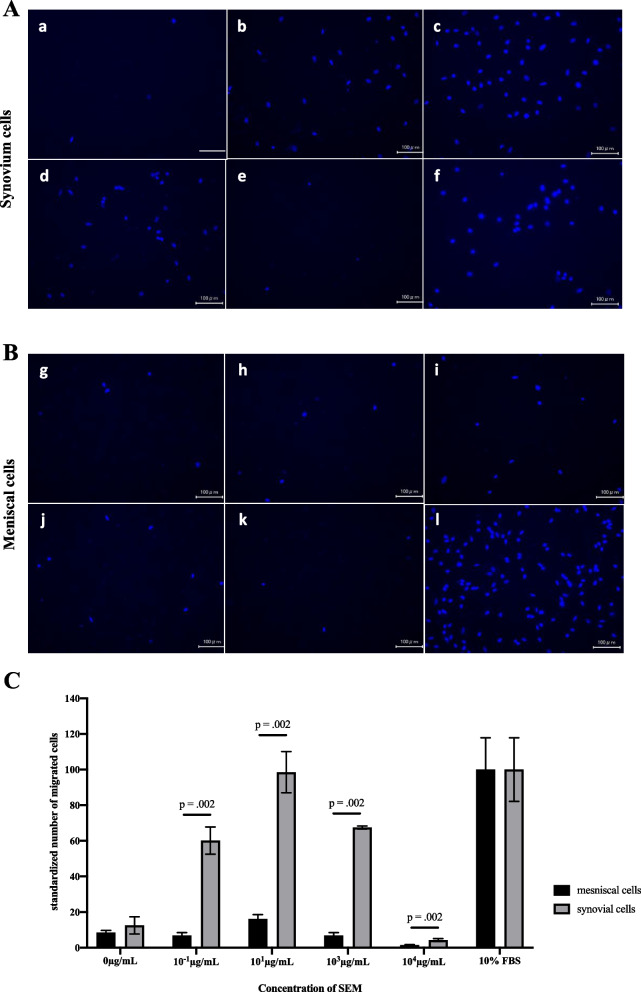
Migration assay using synovial and meniscal cells in the different concentration of SE containing medium (SEM). **A** Synovium cells. **B** Meniscal cells. Bars; 100 μm (**C**) Standardized number of migrated cells

Almost no migration of meniscal cells was confirmed with various concentrations of SEM. On the other hand, in synovial cells, the number of migrating cells changed depending on the concentration of SEM, and the number of cells was the largest at 10 μg/ml of SE, and the number of cells decreased when the concentration was further increased. The migration ability of synovial cells to SE was confirmed (Fig. 2).

### Histological analysis stained with safranin-O / fast green

**Fig. 3:**
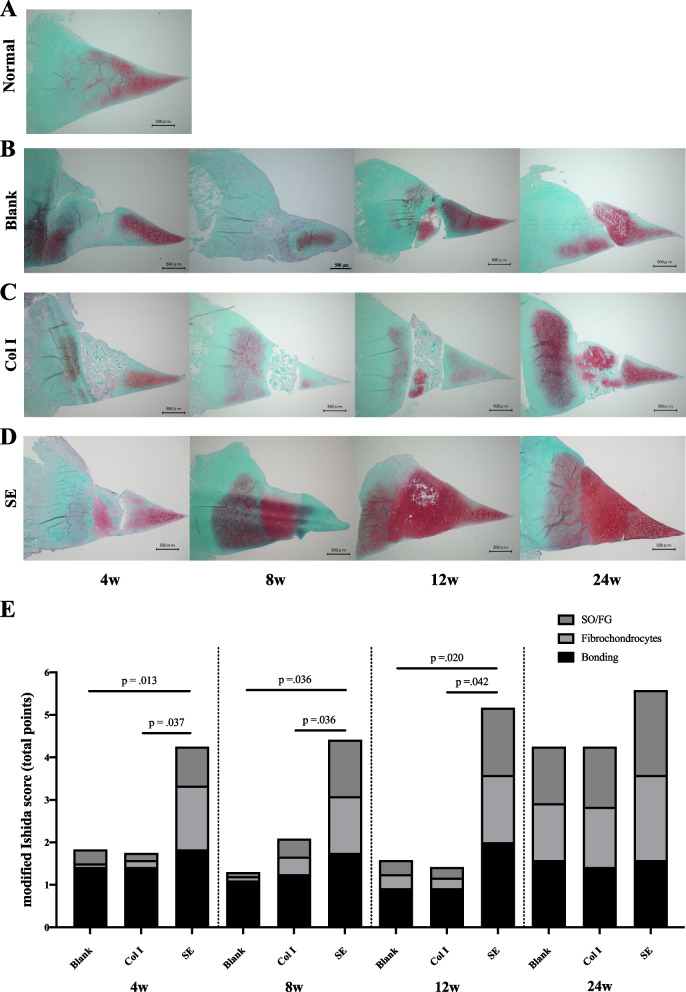
**A**-**D** Safranin-O staining of the medial meniscus in the normal, Blank, Col I and SE groups. Bars: 500 μm. **E** Modified Ishida score. SO/FG: Safranin O / Fast green

The Blank group showed scar-like tissue formation at 4 weeks, although the spread of fibrochondrocytes and staining with safranin-O were poor. At 8 weeks, staining with safranin-O was observed, however, the spread of cells was sparse. At 12 weeks, fibrochondrocytes were partially present and stained densely with safranin-O. The formation of cartilage-like tissue was observed, although there was insufficient bonding to the surrounding tissue and scar-like tissue was still observed. At 24 weeks, the extent of cartilage-like tissue increased and the bonding to the surrounding tissue improved. In the Col I group, the implanted scaffold was still present at 4 weeks, and bonding to the surrounding meniscus was observed, although cell spreading and safranin-O staining were poor. At 8 weeks, the scaffold was absorbed and adhesion was poor, and at 12 weeks, some fibrochondrocytes were confirmed and chondrocyte-like tissue stained with safranin-O at 12 weeks, some fibrocartilage cells were present and cartilage-like tissue stained with safranin-O was formed. However, the bonding to the surrounding tissue was poor. At 24 weeks, the extent of cartilage-like tissue became broader with the replacement of the scaffold and the bonding to the surrounding tissue improved. In the SE group, both fibrochondrocytes and safranin-O staining were already present at 4 weeks. At 8 and 12 weeks, fibrochondrocytes were more widespread and safranin-O staining was more intense. At 24 weeks, fibrochondrocytes were present throughout and safranin-O staining remained intense, however, its staining was reduced compared to that at 12 weeks and still higher level compared to the normal meniscal tissue. The Ishida score was significantly higher in the SE group than in the Blank and Col I groups at 4, 8, and 12 weeks, and there was no significant difference between the Blank and Col I groups. At 24 weeks, there was no significant difference between the SE group, Blank group, and Col I group (Fig. 3).

### Histological analysis stained with HE

**Fig. 4:**
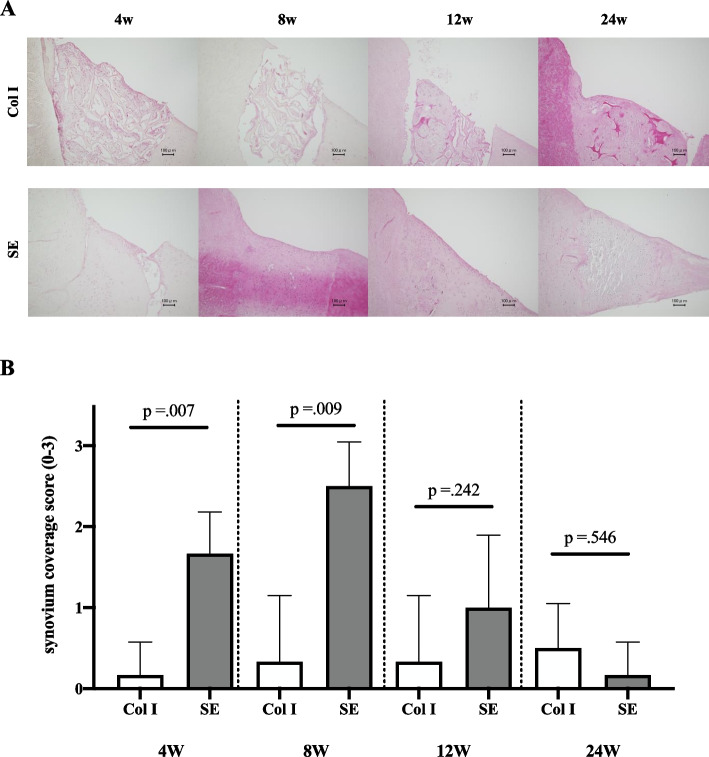
Histology of synovial coverage. **A** Hematoxylin & eosin staining of the medial meniscus in the Col I and SE groups. Bars: 100 μm **B** Synovial coverage score

In the Col I group, 75% of the sections were not covered by synovium, and even the sections that were covered did not have more than 6 layers. In the SE group, all sections had 1–5 layers of synovial cells and 67% of the sections had 3 or more layers at 4 weeks. At 8 weeks, 3 or more layers of synovial cells were present, and 50% of the sections had 6 or more layers. At 12 weeks, the synovial layer had decreased, with no sections having more than 6 layers, and 33% of sections having 0 layers. At 24 weeks, all but one section were not covered with synovial cells, and even the only section was covered with only one layer. The synovial coverage score was significantly higher in the SE group than in the Col I group at 4, and 8 weeks. At 12 and 24 weeks, there was no significant difference between the SE group and Col I group (Fig. 4).

### Immunohistochemical analysis

**Fig. 5:**
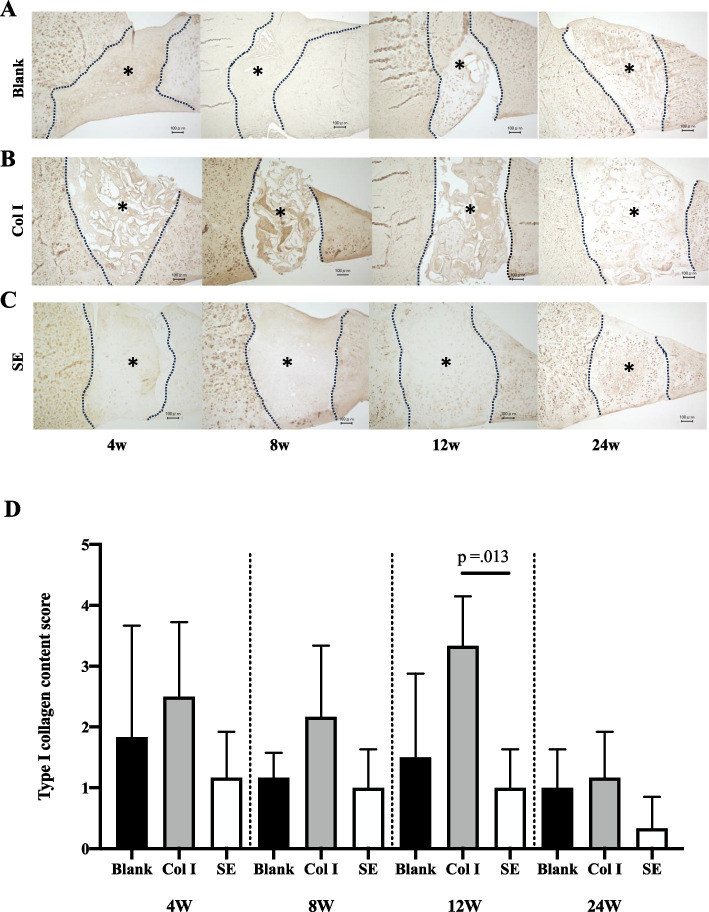
Immunohistochemistry of Col I. **A** Immunostaining for type I collagen in the Blank, Col I and SE groups. *; defect site. Bars; 100 μm (**B**) The type I collagen staining score

Immunostaining for type I collagen showed the average score was highest in the order of the Col I group, the Blank group, and the SE group at all time points. In particular, the difference was greatest at 12 weeks, with 50% of the sections in the Col I group showing staining of over 75%, and the staining score was significantly higher than that in the SE group. At 24 weeks, the staining ratio decreased in all groups, and even in the Col-I group, there were no sections with more than 50% staining, and there was no significant difference among the three groups (Fig. 5).

**Fig. 6:**
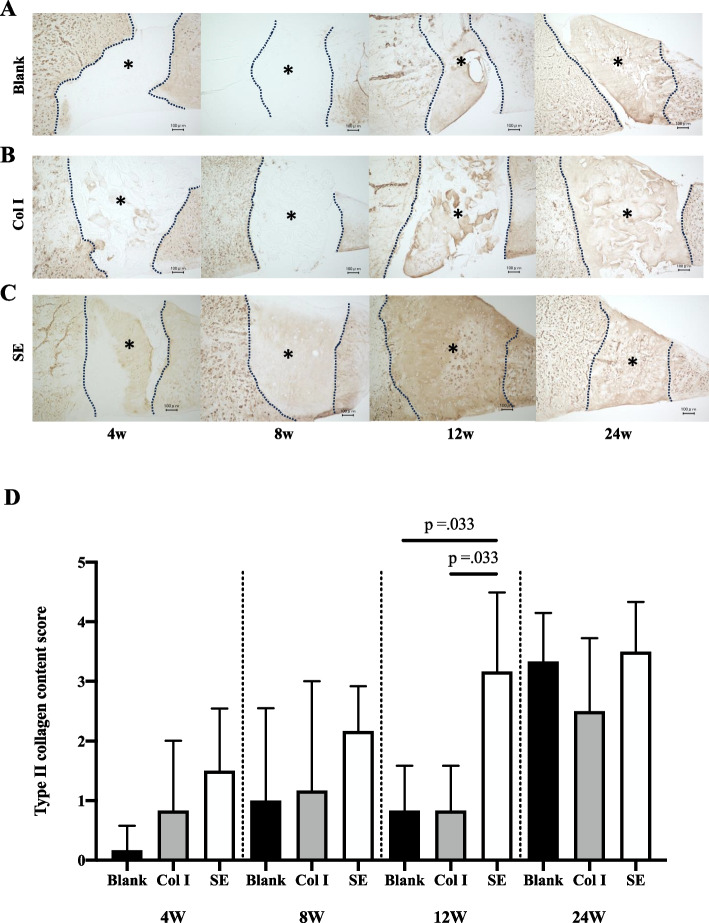
Immunohistochemistry of Col II. **A** Immunostaining for type II collagen in the Blank, Col I and SE groups. *; defect site. Bars; 100 μm (**B**) The type II collagen staining score

**Fig. 7:**
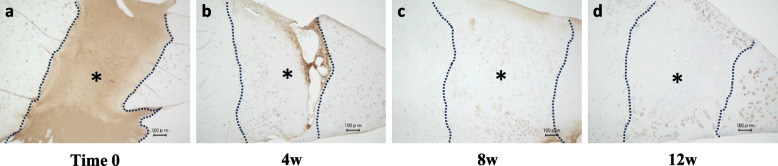
Immunohistochemistry of SE in the SE group. *; defect site. Bars; 100 μm

For type II collagen immunostaining, the SE group had the highest mean score at all time points. Similar to col-I staining, the difference was greatest at 12 weeks. Staining of 75% or higher was observed in 67% of the sections in the SE group, while no staining of 50% or higher was observed in the other two groups, with the SE group having a significantly higher staining score than the Blank and Col I groups. At 24 weeks, the staining rate increased in both the Col-I group and the Blank group, and there was no significant difference among the three groups (Fig. 6).Immunostaining of SE showed localization of SE was observed immediately after transplantation at the defect site. Although SE localization was still presented at 4 weeks, the grafted area had been replaced with safranin-O stained areas. At 8 weeks, it was difficult to confirm SE stained area (Fig. 7).

## Discussion

The present study revealed that SE sponge was biocompatible and could induce good meniscal repair through the promotion of the migration of synovial cells to the injured area in the meniscus in rabbit models. SE promoted superior meniscus repair with fibrocartilage cells, glycosaminoglycan-rich tissue, and type II collagen compared to the control groups (Blank and Col I), which suggests that SE has the potential to be an ideal material for meniscal repair.

Regarding the selection of cell sources for meniscus repair, meniscal cells [33], chondrocytes [34], and mesenchymal stem cells (MSCs) derived from bone marrow [35], fat tissue [36] or synovium [37] have been used. Kim et al. performed freeze–thaw treatment on the meniscus and synovium of Japanese white rabbits to investigate the therapeutic effect of a cylindrical defect of 2 mm-diameter filled with alginate gel. They found that the defects were filled with fibrochondrocytes and proteoglycan when the synovium was intact and concluded that synovial cells were the primary contributors to meniscal injury repair [38]. Nakagawa et al. transplanted synovial MSCs to longitudinal tears in the avascular region of the meniscus in pigs and concluded that synovial MSC transplantation promotes meniscal healing by guiding the synovium to the injured region [31]. In our vitro experiments, synovial cells migrated toward the medium containing SE solution, while meniscal cells did not. In our vivo experiments, almost no synovial cell induction was observed in the Col-I group, while vigorous synovial cell induction was observed in the SE group at 4 and 8 weeks. These results suggest that SE application at the lesion could have the potential to early recruit synovial cells that are a major contributor to meniscal repair. Although the detailed mechanism by which SE promotes synovial cell migration remains to be determined, this feature would be a mode of action of SE for acceleration of meniscal healing.

A semi-quantitative scoring system was developed by Ishida in 2007 to evaluate the regeneration of meniscal tissue in vitro and in vivo after platelet-rich plasma application [29]. Longo et al. [39] validated the histological scoring system and reported that the Ishida score is the most adequate for the evaluation of tissue-engineered meniscal repair, and it has been used in many reports [40–44] of meniscus repair using scaffolds. However, since the scoring is based on three levels (0, 1, and 2), the interpretation of partial repair is ambiguous, and the possible existence of various tissues within the score of 1 was considered to be a problem. Therefore, we modified the tissue scoring to five levels (0, 0.5, 1, 1.5, 2), which could better reflect the tissue repair. Of the three items in the Ishida score, the existence of fibrochondrocytes and staining with safranin-O were particularly early in the SE group, and the tissue matured with time, suggesting that SE contributes to accelerated repair of meniscus injuries in the early stage. Nevertheless, it does not include a specific assessment of the “collagen organization”. Thus, we considered it should be used in association with another score, evaluating the distribution of collagen fibers in the meniscal tissue. Microscopically, the meniscus can be distinguished into a peripheral red zone and a medial, vessel-free white zone [45]. The peripheral zone presents abundant type I collagen deposition and many elongated fibroblast-like cells. In contrast to the peripheral zone, the medial zone is characterized by chondrocyte-like cells embedded in type II collagen and glycosaminoglycans [46, 47]. Zellner et al. developed a scoring system in 2010 for the macroscopic, histological, and immunohistochemical evaluation of avascular meniscal punch defect repair. This scoring system has been used in several reports about meniscal repair [48, 49]. The content of type II collagen is one of the immunohistological items, and is classified into four levels, with the higher the content, the better the repair. We modified the content ratio into five levels (0, 1, 2, 3, 4) and scored each of Type I and II collagen. The Col-I staining rate was generally high in the Col-I group, and the SE group showed high scores for Col-II staining from early after transplantation, suggesting that type II collagen production was promoted along with good tissue repair.

As commercially available materials for meniscal repair, CMI® scaffolds showed poor biomechanical properties, which could lead to iatrogenic damage of the scaffold during implant positioning and suture [50]. To overcome these problems, Actifit® has been developed claiming superior mechanical properties. While intra-operatively this was perceived as a sufficient strength by surgeons, this may be not enough to sustain the high joint stresses, with even weight bearing and simple walking causing numerous and critical stress cycles [8]. Only one prospective, non-randomized trial compared the outcomes of CMI® and Actifit® scaffolds [21]. CMI® was mainly replaced by fibrous tissue, whereas an avascular cartilaginous-like tissue was present in the case of the Actifit®. In all cases, the scaffolds were still visible at the macroscopic and microscopic levels, suggesting an incomplete meniscal healing process after two years from implantation. Thus, more biocompatible material with appropriate mechanical and biological properties has been desired in clinical practice for the successful treatment of meniscal defects. In our study, the implanted SE sponge was efficiently replaced by safranin-O staining positive tissue within 8 weeks, suggesting that residual materials in the meniscus does not interfere with the meniscus healing. In addition, SE sponge has already been clinically applied for chronic skin ulcers and its safety for human has been confirmed [27]. SE has the potential to be an ideal scaffold for meniscal repair in terms of its cell migration ability, ability to provide a three-dimensional framework for tissue repair, and ease of handling. However, although high concentrations of silk elastin improved the ease of handling, it was shown to physically inhibit cell migration, so it is necessary to investigate optimal condition in the future.

There were several limitations to the current study. First, the evaluations of the mechanical strength of repaired tissue and knee joint cartilage wear were not performed in this study. This data will be required for future clinical applications. Second, the SE used in this study does not have enough mechanical properties for suture application due to its hydrophilic nature. Improvement of this mechanical property will be important for applying large meniscal defects to stabilize implanted material with sutures and tolerate mechanical stress during activity until tissue formation. Thirdly, the experiment was conducted in an atypical injury model in clinical practice using rabbits and followed a limited experimental period, a maximum of 6 months. In terms of injury site, we created defect at white-white zone. The effect on the red-red or red-white zones injury models may also need to be investigated. However, as the effect of SE on meniscus repair has been confirmed in white-white zone with poor healing capacity, it may not be possible to fully compare the effect of SE on meniscus repair in red-red or red-white zones where healing is fully expected to be adequate. To confirm the efficacy of SE for meniscal healing and to consider clinical application, large animal experiments, such as the pig model, experiments in injury models that are likely to occur clinically, and longer follow-ups will be required in the near future.

## Conclusion

SE has the property to induce synovial cell migration, but it does not affect meniscal cells. Implantation of SE sponge into meniscal defects accelerates meniscal healing with fibrochondrocytes and glycosaminoglycan- and type II collagen-rich tissues by inducing synovial coverage in rabbits. SE has the potential to be a useful material for meniscal repair.

## Data Availability

All data generated or analyzed during this study are included in this published article.

## Abbreviations

HE: Hematoxylin & eosin
MSC: Mesenchymal stem cell
SE: Silk-elastin
SEM: Silk-elastin containing medium
